# Antibodies elicited by SARS-CoV-2 infection or mRNA vaccines have reduced neutralizing activity against Beta and Omicron pseudoviruses

**DOI:** 10.1126/scitranslmed.abn7842

**Published:** 2022-01-13

**Authors:** Benjamin L. Sievers, Saborni Chakraborty, Yong Xue, Terri Gelbart, Joseph C. Gonzalez, Arianna G. Cassidy, Yarden Golan, Mary Prahl, Stephanie L. Gaw, Prabhu S. Arunachalam, Catherine A. Blish, Scott D. Boyd, Mark M. Davis, Prasanna Jagannathan, Kari C. Nadeau, Bali Pulendran, Upinder Singh, Richard H. Scheuermann, Matthew B. Frieman, Sanjay Vashee, Taia T. Wang, Gene S. Tan

**Affiliations:** ^1^ J. Craig Venter Institute, La Jolla, CA, 92037 and Rockville, MD, 20850 USA.; ^2^ Department of Medicine, Division of Infectious Diseases, Stanford University, Stanford, CA, 94305 USA.; ^3^ Program in Immunology, Stanford University School of Medicine, Stanford, CA, 94305 USA.; ^4^ Division of Maternal-Fetal Medicine, Department of Obstetrics, Gynecology, and Reproductive Sciences, University of California San Francisco, San Francisco, CA, 94115 USA.; ^5^ Department of Bioengineering and Therapeutic Sciences, and Institute for Human Genetics, University of California San Francisco, San Francisco, CA, 94115 USA.; ^6^ Division of Pediatric Infectious Diseases, Department of Pediatrics, University of California, San Francisco, CA, 94115 USA.; ^7^ Institute for Immunity, Transplantation, and Infection, Stanford University School of Medicine, Stanford, CA, 94305 USA.; ^8^ Departments of Pathology and of Microbiology and Immunology, Stanford University School of Medicine, Stanford, CA 94305 USA.; ^9^ Department of Microbiology and Immunology, Stanford University School of Medicine, Stanford, CA, 94305 USA.; ^10^ Howard Hughes Medical Institute, Stanford University School of Medicine, Stanford, CA 94305, USA.; ^11^ Sean N. Parker Center for Allergy and Asthma Research, Department of Medicine, Stanford, CA, 94305 USA.; ^12^ Department of Microbiology and Immunology, Center for Pathogen Research, University of Maryland School of Medicine, Baltimore, MD, 21201 USA.; ^13^ Chan Zuckerberg Biohub, San Francisco, CA, 94158 USA.; ^14^ Division of Infectious Diseases, Department of Medicine, University of California San Diego, La Jolla, CA, 92037 USA.

## Abstract

Multiple severe acute respiratory syndrome coronavirus 2 (SARS-CoV-2) variants that possess mutations associated with increased transmission and antibody escape have arisen over the course of the current pandemic. Although the current vaccines have largely been effective against past variants, the number of mutations found on the Omicron (B.1.1.529) spike protein appear to diminish the protection conferred by pre-existing immunity. Using vesicular stomatitis virus (VSV) pseudoparticles expressing the spike protein of several SARS-CoV-2 variants, we evaluated the magnitude and breadth of the neutralizing antibody response over time in individuals after infection and in mRNA-vaccinated individuals. We observed that boosting increases the magnitude of the antibody response to wildtype (D614), Beta, Delta, and Omicron variants; however, the Omicron variant was the most resistant to neutralization. We further observed that vaccinated healthy adults had robust and broad antibody responses whereas responses may have been reduced in vaccinated pregnant women, underscoring the importance of learning how to maximize mRNA vaccine responses in pregnant populations. Findings from this study show substantial heterogeneity in the magnitude and breadth of responses after infection and mRNA vaccination and may support the addition of more conserved viral antigens to existing SARS-CoV-2 vaccines.

## Teaser

Pre-existing neutralizing antibody activity is diminished against SARS-CoV-2 Omicron spike-expressing pseudoparticles relative to the D164 spike.

## INTRODUCTION

First identified in Botswana in November 2021, the severe acute respiratory syndrome coronavirus 2 (SARS-CoV-2) Omicron variant (B.1.1.529) is rapidly becoming the dominant circulating variant of concern (VOC) (*1*, *2*). The Omicron variant harbors a striking 59 amino acid substitutions throughout its genome relative to the ancestral Wuhan-hu-1 SARS-CoV-2 virus, referred to as D614 here. Thirty-seven of these mutations are within the spike protein, the target of neutralizing antibody responses against this virus. As neutralizing antibodies are the major correlate of protection against coronavirus disease 2019 (COVID-19) (*3*, *4*), this degree of mutational change raises questions about the effectiveness of neutralizing antibodies that were elicited by infection with SARS-CoV-2 (D614) infection or by current mRNA vaccines which encode the D614 spike protein. To define the extent of escape by Omicron from neutralizing antibodies in the population, we evaluated the magnitude and breadth of the response against the D614 virus along with three VOCs, Beta (B.1.351), Delta (B.1.617.2) and Omicron. Understanding these neutralizing antibody responses will enable us to assess the state of pre-existing immunity elicited by the D614 virus and can inform the design of the next generation of COVID-19 vaccines (*5*).

The spike glycoprotein of SARS-CoV-2 has two major antigenic domains; mutations in these regions can contribute to antigenic escape and reduced immunity against infection (*6*). The receptor binding domain (RBD) interacts directly with the receptor for SARS-CoV-2, angiotensin-converting enzyme 2 (ACE2), and amino acid changes in RBD can impact the affinity of spike protein for ACE2 and thus transmissibility and virulence of viral variants. The Beta variant has notable mutations (L18F, D80A, D215G, ∆242-244 and R246I) in the amino terminal domain (NTD) and RBD (K417N, E484K and N501Y) (*7*) of the spike protein that is associated with antibody escape, as previously reported (*8*–*10*). The emergence of the Delta variant, which has mutations in the RBD, resulted in higher transmissibility. This has led to Delta becoming the predominant circulating strain of SARS-CoV-2 until emergence of the Omicron variant. Unlike the Beta variant, the Delta variant has one important mutation in the RBD (L452R) relative to the WT virus that is associated with antibody escape (*4*). Based on these different mutational profiles of the Beta and Delta spike proteins, we chose to include these VOCs in the present study along with the Omicron variant. The Omicron variant, now the dominant circulating SARS-CoV-2 strain, harbors a relative abundance of mutations with 37 non-synonymous changes in the spike protein alone, 11 in the NTD and 15 in the RBD. Based on the structural features of the Omicron spike protein and recent findings by other groups (*11*–*14*), we anticipated that Omicron would be at least as resistant to current neutralizing antibodies in the population as the Beta variant and likely far more resistant compared to the WT and Delta viruses.

To study the relative susceptibility of the spike proteins of SARS-CoV-2 VOC to neutralizing antibodies in the population, we studied activity in serum samples or plasma from three cohorts of previously infected or mRNA-vaccinated individuals against D614, Beta, Delta, and Omicron pseudoviruses. In an infection cohort, we tested plasma collected during the peak phase of mild COVID-19 (day 28 post study enrollment) and two time points during the convalescent period (days 210 and 300 post study enrollment). To understand the breadth of neutralizing antibodies elicited by mRNA vaccination, we studied a cohort of pregnant individuals who received two doses of the Pfizer BNT162b2 or Moderna mRNA-1273 vaccines during pregnancy. Pregnancy is a risk factor for poor outcomes in COVID-19; thus, this cohort provides important insights into immunity in this vulnerable population. A second cohort of healthcare workers received three doses of the Pfizer BNT162b2 vaccine. These approved SARS-CoV-2 mRNA vaccines code for the original D614 SARS-CoV-2 spike protein. Although mRNA vaccines have been extremely successful at inducing potent neutralizing antibody responses, their effectiveness will depend in large part to the degree of antigenic drift in circulating SARS-CoV-2 variants. Using these three distinct cohorts, we evaluated the magnitude and breath of neutralizing antibody titers over time after infection and mRNA vaccination.

## RESULTS

### The magnitude and breadth of the neutralizing antibody response following early pandemic SARS-CoV-2 infections is reduced against the Beta and Omicron variants.

**Fig. 1.**
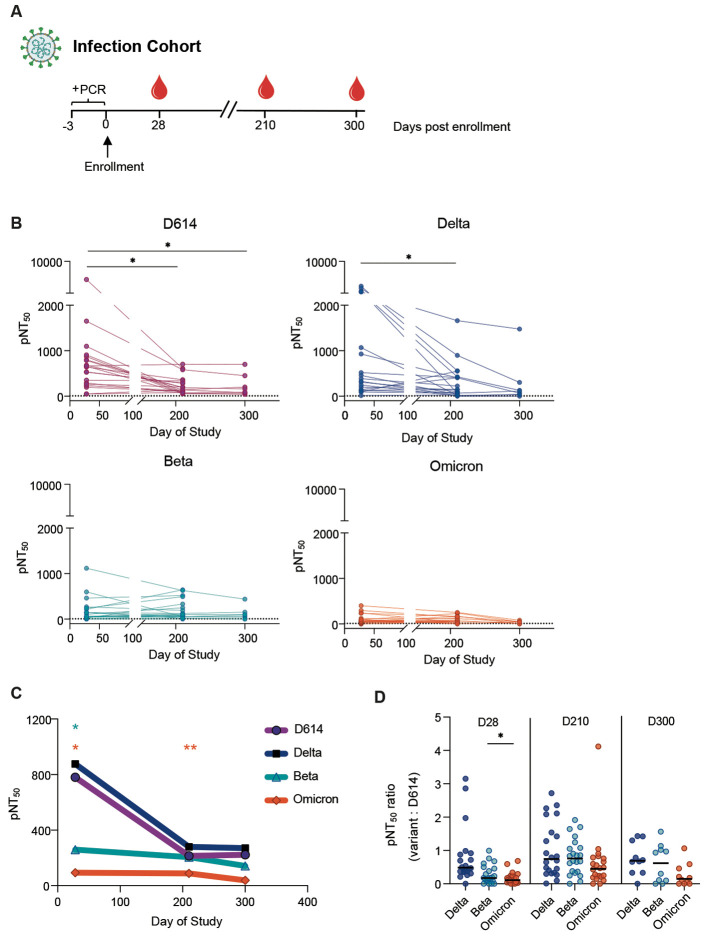
Plasma neutralizing titers against variants of concern wane over time in samples from an outpatient COVID-19 infection cohort. (**A**) Study participants were enrolled (day 0) within three days of a positive SARS-CoV-2 PCR test (+PCR). Longitudinal plasma samples from day 28 (n=23), day 210 (n=23) and day 300 (n=8) were assessed against D614, Delta, Beta and Omicron. (**B**) The kinetics of half-maximal SARS-CoV-2 pseudovirus neutralizing titers (pNT_50_) over time are shown. Dotted lines indicate baseline mean pNT_50_ obtained from seronegative subjects. Solid lines connect samples from the same participant. (**C**) Mean pNT_50_ values are shown across study time-points of all 4 pseudovirus variants. Values that were significantly different from D614 at each time point are marked with a star. (**D**) Ratios of pNT_50_ values of the indicated variants of concern over D614 pNT_50_ at each study time point are shown. Horizontal bars indicate median values. p values in (B to D) were calculated using mixed effects analysis with Geisser-Greenhouse correction and Tukey’s multiple comparisons test. *P < 0.05, **P < 0.01.

We first evaluated a total of 54 plasma samples from three study time-points for neutralizing antibody responses in a group of individuals from a longitudinal cohort of mild SARS-CoV-2 patients enrolled in an outpatient study at Stanford Hospital Center (*15*). This study was a trial evaluating the efficacy of interferon lambda in mild COVID-19, but only participants from the placebo arm have been studied here. Participants in this study were infected with SARS-CoV-2 during the first half of 2020, a time when the D614 virus was the dominant circulating SARS-CoV-2 strain. Three time points were chosen for this analysis: day 28 post enrollment, the previously characterized peak antibody response, and days 210 and 300 post enrollment (**Fig. 1A**) (*16*). Samples from participants who were vaccinated during the study period were excluded from the analysis. Neutralizing antibody titers were highest on day 28 for all VOCs and waned over time in the convalescent period on days 210 and 300 (**Fig. 1B**). Notably, both Beta and Omicron VOC were more resistant to neutralization, even on day 28, as compared to WT and Delta, perhaps reflecting the key mutations found in both the Beta and Omicron spike protein that have been previously described to contribute to antibody escape (*9*). Whereas neutralizing activity against D614 and Delta was measurable in many participants at day 300, activity against the Beta and Omicron variants was largely absent by this later timepoint (**Fig. 1B to D**).

### The magnitude of neutralizing antibody responses against the Omicron variant following mRNA vaccination is reduced, whereas the breadth of responses is highly heterogeneous among vaccine recipients.

**Fig. 2.**
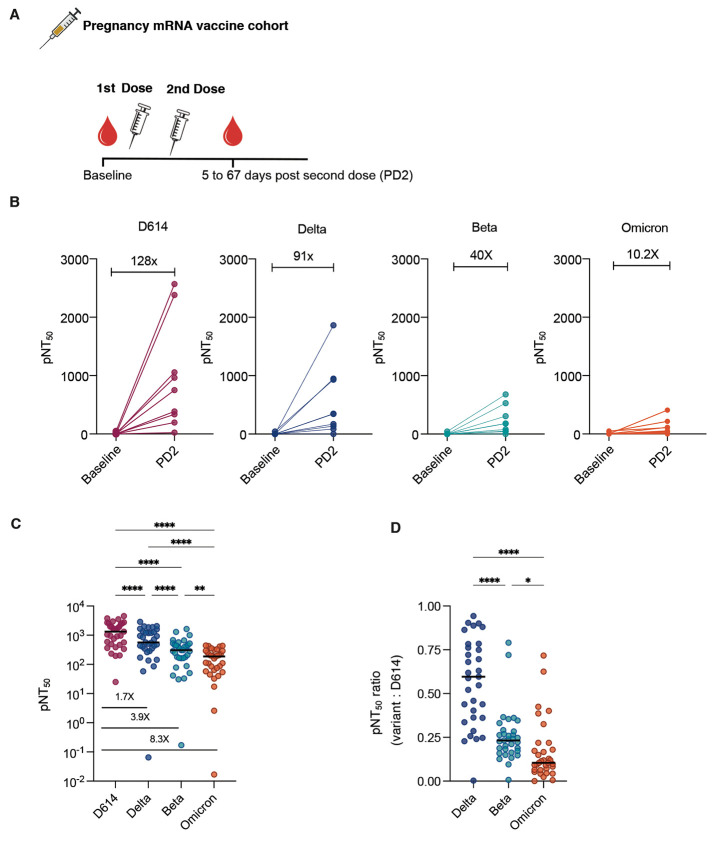
Plasma neutralizing titers against variants of concern increase after mRNA vaccination in a cohort of pregnant women. (**A**) Samples collected from a cohort of vaccinated pregnant women at baseline (n=9) and after 2 doses of mRNA vaccine (n=33) were assessed for neutralizing antibody against 4 SARS-CoV-2 pseudovirus strains. (B) pNT_50_ values of paired samples at baseline and after 2 doses of mRNA vaccine (PD2) are presented. Samples from 9 participants from whom paired samples were available are shown; fold change of mean titers between the 2 time-points are indicated. (**C**) pNT_50_ values against the 4 SARS-CoV-2 pseudoviral variants were measured in samples collected after 2 doses of mRNA vaccine. Fold reduction of mean pNT_50_ compared to D614 are indicated for each variant. (**D**) Ratios of pNT_50_ values of the indicated variants of concern over D614 pNT_50_ are shown. Horizontal bars in (C and D) indicate median values. p values in (C and D) were calculated using repeated measures one way-ANOVA with Geisser Greenhouse correction and with Tukey’s multiple comparisons test. *P < 0.05, **P < 0.01, ****P < 0.0001.

To understand the neutralizing antibody responses elicited by SARS-CoV-2 mRNA vaccines, we studied two separate cohorts of vaccinated individuals. All of the vaccinated participants studied were seronegative for SARS-CoV-2 at baseline. One cohort comprised pregnant individuals that were enrolled in a vaccine study in the University of California San Francisco Health system (n=9 at baseline before vaccination and n=33 following 2 doses of mRNA vaccine) (*17*, *18*). These participants received either the Pfizer BNT162b2 (n=10) or Moderna mRNA-1273 (n=23) mRNA vaccines during pregnancy and the post-vaccination timepoint ranged from 5 to 67 days after dose 2. We tested paired neutralizing antibody responses in a subset of participants (n=9) from serum taken before vaccination (baseline) and in serum collected after the second immunization (post-dose 2, **Fig. 2A**). As expected, the post-dose 2 neutralizing titers were substantial against D614 (the homologous spike protein) after two vaccine doses, with a mean pNT_50_ of about 128-fold over baseline. Response at post-dose 2 in all participants were progressively lower than D614. Specifically, the increase in neutralizing titers against Delta, Beta and Omicron was 91, 40 and 10.2-fold over baseline, respectively (**Fig. 2B**). The magnitude of neutralizing titers against Delta, Beta, and Omicron were all reduced relative to D614 (**Fig. 2C**). Substantial heterogeneity was present among vaccinees in the breadth of neutralizing antibody responses. This was demonstrated in the wide range of ratios displayed by individuals in neutralizing titers against Delta, Beta, and Omicron variants relative to D614 (**Fig. 2D**).

**Fig. 3.**
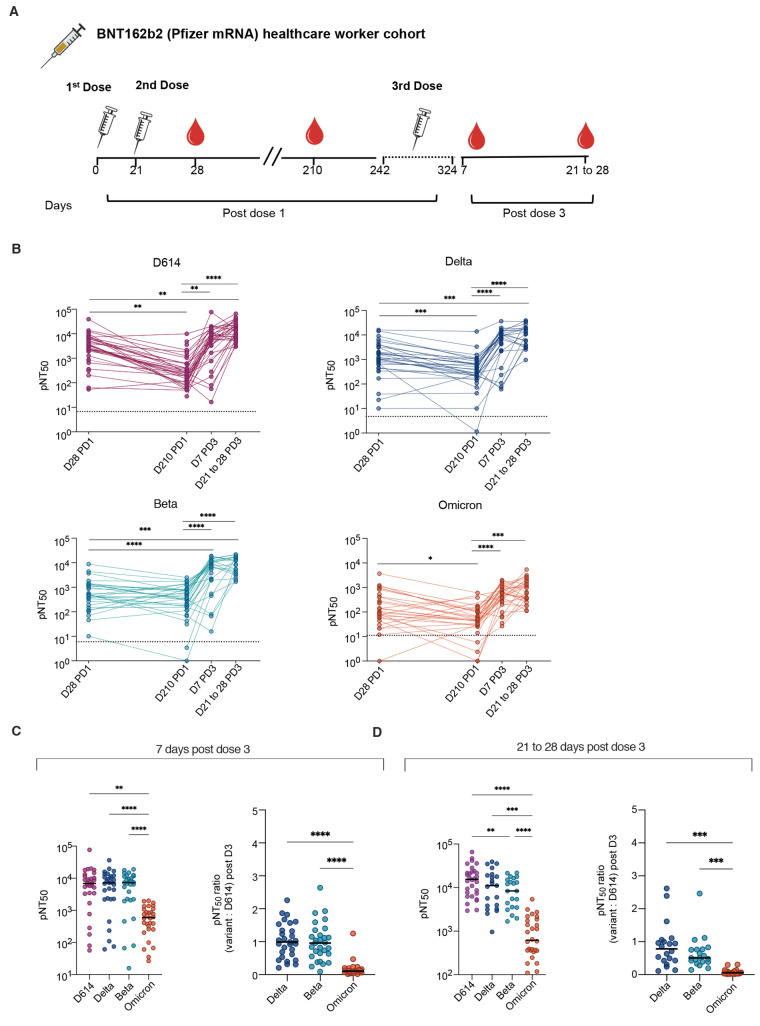
Plasma neutralizing titers against variants of concern are increased after a third dose of mRNA vaccine in a cohort of healthcare workers. (**A**) Samples were collected from a cohort of healthcare workers who received 2 doses of Pfizer vaccine 21 days apart and received a 3^rd^ dose 242 to 324 days after the first dose. Antibody titers were assessed at 28 and 210 days post dose 1 (PD1), and 7 days and 21 to 28 days post dose 3 (PD3). (**B**) The kinetics of pNT_50_ values against D614, Delta, Beta, and Omicron are shown. Dotted lines indicate baseline mean pNT_50_ obtained from seronegative subjects. Solid lines connect samples from the same participant (**C**) pNT_50_ values (left panel) and ratios of pNT_50_ of the indicated variants of concern over D614 pNT_50_ (right panel) are shown for samples collected 7 days after 3^rd^ dose of vaccine. (**D**) pNT_50_ values (left panel) and ratios of pNT_50_ of the indicated variants of concern over D614 pNT_50_ (right panel) are shown for samples collected 21 to 28 days after 3^rd^ dose of vaccine. Horizontal bars in (C and D) indicate median values. p values in (C) were calculated using repeated measures one way-ANOVA with Geisser Greenhouse correction and with Tukey’s multiple comparisons test. p values in (B and D) were calculated using mixed effects analysis with Geisser-Greenhouse correction and Tukey’s multiple comparisons test. *P < 0.05, **P < 0.01, ***P < 0.001, ****P < 0.0001.

Finally, we evaluated the neutralizing antibody responses from a cohort of healthcare workers previously vaccinated with the Pfizer BNT162b2 mRNA vaccine at the Stanford Hospital Center (n=137 samples) (*19*, *20*). Four time points were chosen for this analysis. An early timepoint following the second dose (seven days post dose 2 or study day 28), followed by a late timepoint at study day 210 prior to dose 3 enabled us to study the durability of neutralizing responses after 2 doses of vaccine. In addition, we defined neutralizing responses on day 7 or between day 21 and 28 post dose 3 (**Fig. 3A**). Neutralizing antibody titers against D614 and all variants were generally highest at 7 days after dose 2 or dose 3. As expected, titers had waned substantially against all variants by day 210 after dose 2. The third vaccine dose increased the titers against all variants substantially, such that they matched, and in most cases surpassed, titers observed at 7 days post dose 2 (**Fig. 3B**). By days 21 to 28 after dose 3, some individuals already had reduced neutralizing antibody titers, whereas others maintained durable titers, approximating those observed on 7 days post dose 3. The antibody titers against Omicron were measurable in all participants after dose 3 but were lower than other variants (**Fig. 3C and D**). As in the cohort of pregnant individuals, substantial heterogeneity was observed in the breadth of the neutralizing antibody responses, as shown by the ratio of titers against variants compared to the titers against D614 (**Fig. 3C and D**).

## DISCUSSION

Using three longitudinal cohorts, we measured the magnitude and breath of the antibody response against D614, Delta, Beta, and Omicron SARS-CoV-2 pseudoparticles in serum isolated from participants following infection or mRNA vaccination. Infection during the early pandemic, when D614 was the dominant circulating SARS-CoV-2 virus, elicited a relatively low neutralizing antibody response against Beta and Omicron variant pseudoviruses, whereas immunity against Delta variant was not reduced over D614. There was clear heterogeneity in this cohort with respect to both the magnitude and breadth of neutralizing responses against all variants tested. In two vaccine cohorts, we observed robust responses elicited by mRNA SARS-CoV-2 vaccines. In all three cohorts, responses against Beta and Omicron pseudoviruses were reduced relative to the homologous D614 and Delta, as has been previously described (*5*–*7*). In a cohort of healthcare workers where we were able to evaluate responses after 3 vaccine doses, there was clearly a benefit to both magnitude and breadth of the response conferred by the third dose. Most importantly, titers against the Omicron pseudovirus were robust after dose 3 relative to dose 2. There was substantial heterogeneity in responses, particularly with respect to breadth, after 2 and 3 doses of mRNA vaccines. Determinants of broadly neutralizing antibody responses after homologous vaccine boosts are not well understood at this time and are an important topic to address in future studies.

We note that this study has limitations, one of which involves the use of pseudotyped viral particles in the neutralization assays. While studies have demonstrated that assays performed with pseudotyped viruses correlate with those performed with wildtype viruses, some pseudotyped viruses are more sensitive to neutralization (*21*, *22*). Second, while neutralizing antibody titers are a major correlate of protective immunity against COVID-19, they are not the only determinant of immunity. Non-neutralizing antibody responses (*23*), T cell responses (*24*, *25*), and innate immune responses (*26*) also impact the outcome of SARS-CoV-2 infections and those parameters have not been studied here. Third, the timing of post-vaccination blood draws was not perfectly matched between the pregnant and non-pregnant cohorts, precluding a direct comparison between responses. Because COVID-19 is a risk factor for adverse outcomes in pregnancy (*27*), it is critical to understand the response to mRNA vaccines in this population and follow-up studies designed to compare the response in pregnancy with non-pregnant cohorts are warranted. Here, we show a relatively consistent response in the magnitude of response after two vaccine doses in pregnancy but note that, although timepoints were not exactly matched with those in the healthy vaccine cohort, the titers were generally lower in samples collected from pregnant individuals. Understanding optimal timing during pregnancy for booster doses will be important for protecting this population (*28*).

Most approved vaccines rely solely on eliciting immune responses against a single immunogen, the spike protein. Although clinical trials have demonstrated excellent effectiveness of mRNA vaccines up to now, it is evident that they will likely have somewhat reduced effectiveness against variants that are antigenically drifted to the extent of the Omicron variant. As SARS-CoV-2 evolves under the selective pressure of neutralizing antibodies, vaccines that rely solely on eliciting responses against the spike protein will also require updating. Supplementing spike protein-based vaccines with other more conserved viral antigens would likely elicit greater breadth of immunity and likely enable less frequent updating of SARS-CoV-2 vaccines (*29*).

## MATERIALS AND METHODS

### Study Design

The goal of this study was to define the neutralizing antibody titers against the WT (D614), Beta, Delta, and Omicron variants of SARS-CoV-2 after SARS-CoV-2 infection or mRNA vaccination. Three cohorts were studied: a longitudinal cohort of patients enrolled with mild COVID-19 (*15*), a cohort of pregnant women who were vaccinated with an mRNA SARS-CoV-2 vaccine during pregnancy (*17*, *18*), and a cohort of adults who received the Pfizer mRNA SARS-CoV-2 vaccine (*19*). Neutralizing antibody titers were studied using a vesicular stomatitis virus (VSV)-based pseudoparticle assay. Data from all available samples from each time point studied are shown. All experiments were performed in duplicate between 1 and 3 times.

Characterization of these samples at Stanford was performed under a protocol approved by the Institutional Review Board of Stanford University (protocol #55718). A subset of available plasma samples (as indicated in the main text) from all the cohorts were used in this study. For the Stanford Lambda cohort, 120 participants were enrolled in a phase 2 randomized controlled trial of Peginterferon Lambda-1a (Lambda, NCT04331899) Inclusion/exclusion criteria and the study protocol for the trial have been published (*12*). Briefly, adults aged 18 to 75 years with an FDA emergency use authorized reverse transcription-polymerase chain reaction (RT-PCR) positive for SARS-CoV-2 within 72 hours prior to enrollment were eligible for study participation. Exclusion criteria included hospitalization, respiratory rate >20 breaths per minute, room air oxygen saturation <94%, pregnancy or breastfeeding, decompensated liver disease, recent use of investigational or immunomodulatory agents for treatment of COVID-19, and prespecified lab abnormalities. All participants gave written informed consent, and all study procedures were approved by the Institutional Review Board of Stanford University (IRB-55619). Participants were randomized to receive a single subcutaneous injection of Lambda or saline placebo. Peripheral blood was collected at enrollment, day 5, and day 28 post enrollment. A subset of participants (n=80) returned for long-term follow-up visits 4-, 7-, and 10-months post enrollment, with peripheral blood obtained. Longitudinal samples from the 56 SARS-CoV-2-infected outpatients who were in the placebo arm of the broader Lambda study were obtained and assessed here.

For the UCSF pregnancy vaccine cohort, 58 female pregnant volunteers that received either the Moderna or Pfizer vaccine were enrolled in a study approved by the University of California at San Francisco Institutional Review Board (20-32077). Plasma samples were collected up to 24 hours before first vaccine dose, up to 24 hours before second dose, and 4 to 8 weeks after second vaccine dose. The median age at enrollment was 35 years old (range 27 to 42). The median gestational age was 21.5 weeks (range 5 to 40) (*17*, *18*).

For the Stanford adult vaccine cohort, 57 healthy volunteers were enrolled in the study approved by Stanford University Institutional Review Board (IRB 8629). The median age was 36 years old with a range from 19 to 79 years old. There were 28 males and 29 females in the study. There were 27 White participants, 23 Asian participants, 4 Black participants, 1 Native American participant, and 2 other participants.

### SARS-CoV-2 variant spike gene construction

The D614 SARS-CoV-2 spike gene was previously amplified with KOD Xtreme Hot Start DNA polymerase (Millipore-Sigma) using cDNA from SARS-CoV-2/human/USA/WA-CDC-WA1/2020 (GenBank MN985325.1) and cloned into pCC1BAC-his3 vector (*30*). To generate SARS-CoV-2 variants’ spike genes, fragments were amplified with Platinum SuperFi II DNA polymerase (Thermo Fisher Scientific) using WT spike plasmid as a template. Desired mutations (table S1) were introduced by primers to each amplicon which has 30 to 35 bp homologous sequences at each end to the adjacent fragments. These amplicons were digested with DpnI (New England Biolabs) to remove template DNA and purified by Qiagen PCR purification kit. Fifty fmol of each amplicon and 15 fmol of YCP/BAC vector were covalently joined using standard Gibson assembly reaction (New England Biolabs), transformed into *E.coli* DH10B competent cells (Thermo Fisher Scientific), and plated on LB medium with 12.5 mg/ml chloramphenicol. *E.coli* transformants were verified to contain correct mutations using PCR and Sanger sequencing (GeneWiz). Plasmids were isolated from *E. coli* by the Purelink HiPure Plasmid Midiprep Kit (Thermo Fisher Scientific). Primers used for spike gene construction and verification are listed in table S2. Lastly, the spike genes lacking the cytoplasmic domain by deleting the last 18 amino acids (S∆18) were then cloned into the pCAGGS expression vector.

**Generation of SARS-CoV-2 pseudoparticles.** To generate VSV pseudotyped with SARS-CoV-2 spike protein, we first coated 6-well plates with 50 μg/mL poly-D-lysine (Thermo Fisher Scientific, Cat. No. A3890401) for 1 to 2 hours at room temperature. After poly-D-lysine treatment, plates were washed three times with sterile water and then seeded with 1.5x10^6^ HEK 293T (American Type Culture Collection, CRL-3216) cells per well. After 24 hours, cells were transfected with 1 μg of pCAGGS-S∆18 per well using Lipofectamine 2000 transfection reagent (Thermo Fisher Scientific, Cat. No. 11668019). Forty-eight hours after transfection, the cells were washed once with 1X phosphate-buffered saline (PBS) and were infected with VSV-∆G-GFP/nanoluciferase or VSV-∆G-RFP/nanoluciferase (a generous gift from Matthias J. Schnell) at a multiplicity of infection of 2 in a 500 μL volume. Cells were infected for an hour with intermittent rocking every 15 min. After infection, the inoculum was carefully removed, and the cell monolayer was washed three times with 1X PBS to remove residual VSV-∆G-GFP/nanoluciferase. Two mL of infection media (2% fetal bovine serum, 1% glutamine, 1% sodium pyruvate, 1% non-essential amino acids and 1% penicillin/streptomycin in 1X Dulbecco's Modified Eagle Medium) was added to each well. At 24 hours post-infection, the supernatants from all the wells were combined and centrifuged (2075 *g* for 30 min, 4°C), and stored at -80°C until use.

**Neutralization assays.** Vero E6-TMPRSS2-T2A-ACE2 (obtained from BEI Resources, NIAID; NR-54970) were seeded at 5x10^5^ cells per well in 50 μL aliquots in half area Greiner 96-well plates (Greiner Bio-One; Cat. No. 675090) 24 hours prior to performing the neutralization assay. On separate U-bottom plates, participant plasma was plated in duplicates and serially 5-fold diluted in infection media for a final volume of 28 μL per well. We also included ‘virus only’ and ‘media only’ controls. Twenty-five microliters containing about 250 to 500 fluorescent forming units (FFUs) of a VSV encoding eGFP gene pseudotyped with one spike protein variant and about 250 to 500 FFUs of a second VSV encoding an mCherry red gene pseudotyped with another spike protein variant was added to each well and incubated at 37°C. Prior to infection, Vero E6-TMPRSS2-T2A-ACE2 cells were washed with 1X PBS. Then 50 μL of the incubated pseudotyped particles and participant plasma mixture was then transferred from the U-bottom 96-well dilution plates onto the monolayer and placed into an incubator at 37°C and 5% CO2. At 17 to 24 hours post-incubation, the number of GFP- and RFP-expressing cells indicating viral infection were quantified using a Celigo Image Cytometer (Nexcelcom Bioscience). We first calculated the percent infection based on our ‘virus only’ controls and then calculate percent inhibition by subtracting the percent infection from 100. A non-linear curve and pNT_50_ values were generated using GraphPad Prism.

**Statistics**. Statistical significance of the data was performed in GraphPad Prism version 9 (GraphPad Software). Either repeated measures one-way analysis of variance (RM-ANOVA) or mixed-effects analysis, both with Geisser Greenhouse correction was used to calculate significance, depending on the samples, with Tukey’s correction for multiple comparisons. The analysis methods applied for each figure are stated in the legends.

## Supplementary Materials

### Other Supplementary Material for this manuscript includes the following:

Data file S1

MDAR Reproducibility Checklist
