# Supplementary material for: Antibodies elicited by SARS-CoV-2 infection or mRNA vaccines have reduced neutralizing activity against Beta and Omicron pseudoviruses
Source: Sci Transl Med. 2022 Jan 13:eabn7842. doi: 10.1126/scitranslmed.abn7842 (PMC8891085; doi:10.1126/scitranslmed.abn7842)
Supplement: Supplementary file 1 — Table S1 [file scitranslmed.abn7842_sm.pdf]

Science Translational Medicine
AAAS

Supplementary Materials for

Antibodies elicited by SARS-CoV-2 infection or mRNA vaccines have reduced neutralizing activity against Beta and Omicron pseudoviruses

Benjamin L. Sievers *et al.*

Corresponding authors: Taia T. Wang, taiawang@stanford.edu; Gene S. Tan, gtan@jcvi.org

DOI: 10.1126/scitranslmed.abn7842

The PDF file includes:

Table S1

Other Supplementary Material for this manuscript includes the following:

Data file S1
MDAR Reproducibility Checklist

Supplemental tables

Table S1. Variant spike protein mutations.

**Lineage**	**Spike protein amino acid substitutions versus WA1**
B.1.351	L18F, D80A, D215G, R246I, K417N, E484K, N501Y, D614G, A701V
B.1.617.2	T19R, G142D, E156-, F157-, R158G, L452R, T478K, D614G, P681R, D950N
B.1.529	A67V, H69-, V70-, T95I, G142D, V143-, Y144-, Y145-, N211-, L212I, ins214EPE, G339D, S371L, S373P, S375F, K417N, N440K, G446S, S477N, T478K, E484A, Q493R, G496S, Q498R, N501Y, Y505H, T547K, D614G, H655Y, N679K, P681H, N764K, D796Y, N856K, Q954H, N969K, L981F,

Table S2. Primer sequences used for this study.

**Name**	**Sequence**	**Description**
ConSR	TTGTTTTCTCTAATTATAAGTCTACCTTTACTAAGAAGAGTAGGGATAACAGGGTAATCGGGTACCGAGCTCGAATTC	SARS-CoV-2 spike pCC1-his vector construction primers
ConSF	AACTGTAACTTTGAAGCAAGGTGAAATCAAGGATGCTACTATTACCCTGTTATCCCTAGATCCTCTAGAGTCGACCTG	
S-F	CTCTTCTTAGTAAAGGTAGACTTATAA	Primers to generate B.1.135 spike gene fragments
SA_C21614T_R	GAGTTCTGGTTGTAAAATTAACACACTGACT	
SA_C21614T_F	AGTCAGTGTGTTAATTTTACAACCAGAACTC	
SA_A21801C_R	GGTAGGACAGGGTTAGCAAACCTCTTAGTAC	
SA_A21801C_F	GTACTAAGAGGTTTGCTAACCCTGTCCTACC	
SA_A22206G_R	AAACCCTGAGGGAGACCACGCACTAAATTAA	
SA_A22206G_F	TTAATTTAGTGCGTGGTCTCCCTCAGGGTTT	
SA_G22299T_R	GGAGTCAAATAACTTATATGTAAAGCAAGTA	
SA_G22299T_F	TACTTGCTTTACATATAAGTTATTTGACTCC	
SA_G22813T_R	ATTATAATCAGCAATATTTCCAGTTTGCCCT	
SA_G22813T_F	AGGGCAAACTGGAAATATTGCTGATTATAAT	

SA_G23012A_R	AACAATTAAAACCTTTAACACCATTACAAGG	
SA_G23012A_F	CCTTGTAATGGTGTTAAAGGTTTTAATTGTT	
UK&SA_A23063T_R	GGTAACCAACACCATAAGTGGGTTGGAAACC	
UK&SA_A23063T_F	GGTTTCCAACCCACTTATGGTGTTGGTTACC	
SA_A23403G_R	TCTGTGCAGTTAACACCCTGATAAAGAACAG	
SA_A23403G_F	CTGTTCTTTATCAGGGTGTTAACTGCACAGA	
SA_C23664T_R	GCAACTGAATTTTCTACACCAAGTGACATAG	
SA_C23664T_F	CTATGTCACTTGGTGTAGAAAATTCAGTTGC	
S_3000_R	ATTTGCACTTCAGCCTCAAC	
S_3000_F	TCCTTTCACGTCTTGACAAA	
S-R	AGTAGCATCCTTGATTTCACCTTG	
S-F	CTCTTCTTAGTAAAGGTAGACTTATAA	Primers to generate B.1.617.2 spike gene fragments
In_T19R_R	AATTGAGTTCTGGTTCTAAGATTAACACACT	
In_T19R_F	AGTGTGTTAATCTTAGAACCAGAACTCAATT	
In_G142D_R	CACTTTCCATCCAACTTTTGTTGTTTTTGTGGTAATAAACATCCAAAAATGGATCAT	
In_E156_R158G_F	ACAAAAACAACAAAAGTTGGATGGAAAGTGGAGTTTATTCTAGTGCG	
In_L452R_R	GATAGATTTCAGTTGAAATATCTCTCTCAAAAGGTTTGAGATTAGACTTCCTAAACAATCTATACCGGTAATTATAATTAC	
In_T478K_F	TTTTGAGAGAGATATTTCAACTGAAATCTATCAGGCCGGTAGCAAACCTTGTAATGGTGT	
SA_A23403G_R	TCTGTGCAGTTAACACCCTGATAAAGAACAG	
SA_A23403G_F	CTGTTCTTTATCAGGGTGTTAACTGCACAGA	
In_L681R_R	CTACGTGCCCGCCGACGAGAATTAGTCTGAG	
In_L681R_F	CTCAGACTAATTCTCGTCGGCGGGCACGTAG	
In_D950N_R	TTTGGTTGACCACATTTTGAAGTTTTCCAAG	
In_D950N_F	CTTGGAAAACTTCAAAATGTGGTCAACCAAA	
S-R	AGTAGCATCCTTGATTTCACCTTG	

S-F	CTCTTCTTAGTAAAGGTAGACTTATAA	Primers to generate B.1.1.529 spike gene fragments
A67VH69V70del_R	CCATTGGTCCCAGAGATAACATGGAACCAAGTAA	
A67VH69V70del_F	TTACTTGGTTCCATGTTATCTCTGGGACCAATGG	
T95I_R	ATGTTAGACTTCTCAATGGAAGCAAAATAAA	
T95I_F	TTTATTTTGCTTCCATTGAGAAGTCTAACAT	
G142DV143Y144Y145del_R	TGTTGTTTTTGTGGTCCAAAAATGGATCAT	
G142DV143Y144Y145del_F	ATGATCCATTTTTGGACCACAAAAACAACA	
N211del212Iins214EPE_R	AAACCCTGAGGGAGATCTTCTGGCTCACGCACTATAATAGGCGTGTGCT	
N211del212Iins214EPE_F	AGCACACGCCTATTATAGTGCGTGAGCCAGAAGATCTCCCTCAGGGTTT	
G339D_R	GCGTTAAAAACTTCATCAAAAGGGCACAAGT	
G339D_F	ACTTGTGCCCTTTTGATGAAGTTTTTAACGC	
S371LS373PS375F_R	TAACACTTAAAAGTGAAAAATGGTGCGAGATTATATAGGACAGA	
S371LS373PS375F_F	TCTGTCCTATATAATCTCGCACCATTTTTCACTTTTAAGTGTTA	
SA_G22813T_R	ATTATAATCAGCAATATTTCCAGTTTGCCCT	
SA_G22813T_F	AGGGCAAACTGGAAATATTGCTGATTATAAT	
N440KG446S_R	AATTATAATTACCACTAACCTTAGAATCAAGCTTGTTAGAATTCCAA	
N440KG446S_F	TTGGAATTCTAACAAGCTTGATTCTAAGGTTAGTGGTAATTATAATT	
S477NT478K_R	TAACAATTAAAACCTGCAACACCATTACAAGGTTTGTTACCGGCCTGATAGA	
S477N-Y505H_F	ACAAACCTTGTAATGGTGTTGCAGGTTTTAATTGTTACTTTCCTTTACGATCATATAGTTTCCGACCCACTTATGGTGTTGGTCACCAACCATACAGAGT	

T547K_R	AGAACACCTGTGCCTTTTAAACCATTGAAGT	
T547K_F	ACTTCAATGGTTTAAAAGGCACAGGTGTTCT	
SA_A23403G_R	TCTGTGCAGTTAACACCCTGATAAAGAACAG	
SA_A23403G_F	CTGTTCTTTATCAGGGTGTTAACTGCACAGA	
H655Y_R	ATGAGTTGTTGACATATTCAGCCCCTATTAA	
H655Y_F	TTAATAGGGGCTGAATATGTCAACAACTCAT	
N679KP681H_R	CTACGTGCCCGCCGATGAGACTTAGTCTGAGTCTGA	
N679KP681H_F	TCAGACTCAGACTAAGTCTCATCGGCGGGCACGTAG	
N764K_R	TCCAGTTAAAGCACGTTTTAATTGTGTACAA	
N764K_F	TTGTACACAATTAAAACGTGCTTTAACTGGA	
D796Y_R	TAAAACCACCAAAATATTTAATTGGTGGTGT	
D796Y_F	ACACCACCAATTAAATATTTTGGTGGTTTTA	
N856K_R	CAAAACAGTAAGGCCTTTAAACTTTTGTGCA	
N856K_F	TGCACAAAAGTTTAAAGGCCTTACTGTTTTG	
Q954H_R	TTAACAAGCGTGTTTAAAGCTTGTGCATTATGGTTGACCACATCT	
Q954HN696KL981F_F	TAATGCACAAGCTTTAAACACGCTTGTTAAACAACTTAGCTCCAAATTTGGTGCAATTTCAAGTGTTTTAAATGATATCTTTTCACGTCTTGACAAAGTT	
S-R	AGTAGCATCCTTGATTTCACCTTG	
RCO495	ACGACGGCCAGTGAATTG	Detection PCR primers to screen for positive clones
Hu1-24a-R	TCTGTAATGGTTCCATTTTC	
Hu1-24b-F	GGTGCTGCAGCTTATTATGT	
Hu1-24b-R	ACCGGCCTGATAGATTTCAG	
S-1200-F	TCAGACAAATCGCTCCAGGGCA	
S-2600-R	CACAAATGAGGTCTCTAGCA	
Hu1-26-F	GGCAGTTTTTGTACACAATT	
Hu1-26-R	GAAAGTGTGCTTTTCCATCA	

Hu1-27-F	GCATGTGACTTATGTCCCTG	
RCO493	GTCTCACCTAAATAGCTTGG	
